# Hepatoma-derived growth factor/nucleolin axis as a novel oncogenic pathway in liver carcinogenesis

**DOI:** 10.18632/oncotarget.3608

**Published:** 2015-04-17

**Authors:** San-Cher Chen, Tsung-Hui Hu, Chao-Cheng Huang, Mei-Lang Kung, Tian-Huei Chu, Li-Na Yi, Shih-Tsung Huang, Hoi-Hung Chan, Jiin-Haur Chuang, Li-Feng Liu, Han-Chung Wu, Deng-Chyang Wu, Min-Chi Chang, Ming-Hong Tai

**Affiliations:** ^1^ Center for Neuroscience, National Sun Yat-Sen University, Kaohsiung 804, Taiwan; ^2^ Division of Hepato-Gastroenterology, Chang Gung Memorial Hospital-Kaohsiung Medical Center, Chang Gung University College of Medicine, Kaohsiung 833, Taiwan; ^3^ Department of Pathology, Chang Gung Memorial Hospital-Kaohsiung Medical Center, Chang Gung University College of Medicine, Kaohsiung 833, Taiwan; ^4^ Department of Chemistry, National Sun Yat-Sen University, Kaohsiung 804, Taiwan; ^5^ Institute of Biomedical Sciences, National Sun Yat-Sen University, Kaohsiung 804, Taiwan; ^6^ Graduate Program in Marine Biotechnology, National Sun Yat-Sen University, Kaohsiung 804, Taiwan; ^7^ Division of Gastroenterology, Department of Internal Medicine, Kaohsiung Veterans General Hospital, Kaohsiung 813, Taiwan; ^8^ Department of Pediatric Surgery, Chang Gung Memorial Hospital-Kaohsiung Medical Center, Chang Gung University College of Medicine, Kaohsiung 833, Taiwan; ^9^ Department of Biological Science & Technology, I-Shou University, Kaohsiung 840, Taiwan; ^10^ Institute of Cellular and Organismic Biology and Genomics Research Center, Academia Sinica, Taipei 115, Taiwan; ^11^ Center for Stem Cell Research and Division of Gastroenterology, Department of Internal Medicine, Kaohsiung Medical University, Kaohsiung 807, Taiwan; ^12^ Division of Colorectal Surgery, Department of Internal Medicine, Kaohsiung Veterans General Hospital, Kaohsiung 813, Taiwan

**Keywords:** hepatoma-derived growth factor, nucleolin, hepatocellular carcinoma, tumour progression

## Abstract

Hepatoma-derived growth factor (HDGF) overexpression is involved in liver fibrosis and carcinogenesis. However, the receptor(s) and signaling for HDGF remain unclear. By using affinity chromatography and proteomic techniques, nucleolin (NCL) was identified and validated as a HDGF-interacting membrane protein in hepatoma cells. Exogenous HDGF elicited the membrane NCL accumulation within 0.5 hour by protein stabilization and transcriptional NCL upregulation within 24 hours. Blockade of surface NCL by antibodies neutralization potently suppressed HDGF uptake and HDGF-stimulated phosphatidylinositol 3-kinase (PI3K)/Akt signaling in hepatoma cells. By using rescectd hepatocellular carcinoma (HCC) tissues, immunohistochemical analysis revealed NCL overexpression was correlated with tumour grades, vascular invasion, serum alpha-fetoprotein levels and the poor survival in HCC patients. Multivariate analysis showed NCL was an independent prognostic factor for survival outcome of HCC patients after surgery. To delineate the role of NCL in liver carcinogenesis, ectopic NCL overexpression promoted the oncogenic behaviours and induced PI3K/Akt activation in hepatoma cells. Conversely, NCL knockdown by RNA interference attenuated the oncogenic behaviours and PI3K/Akt signaling, which could be partially rescued by exogenous HDGF supply. In summary, this study provides the first evidence that surface NCL transmits the oncogenic signaling of HDGF and facilitates a novel diagnostic and therapeutic target for HCC.

## INTRODUCTION

Hepatocellular carcinoma (HCC) is the fifth most common cancer and the third leading cause of cancer death worldwide [1]. Early stage HCC is frequently asymptomatic that most HCC patients are diagnosed at intermediate or advanced stages. Despite the recent advances in biomedical sciences, the molecular mechanism underlying liver carcinogenesis remains far from elucidated. Hence, a major clinical challenge is to identify the novel molecular markers for HCC diagnosis and therapy.

Hepatoma-derived growth factor (HDGF) is a protein of 240 amino-acid isolated from the cultured supernatants of human hepatoma cells [2]. HDGF is composed of a highly conserved N-terminal 100 residues HATH (homologous to the amino terminus of HDGF) domain and a variable C-terminal 140 residues (C140) domain. With two bipartite nuclear localization sequences, HDGF is mainly localized in nucleus and stimulates the proliferation in various types of cells including fibroblasts, endothelial cells and hepatoma cells [3]. HDGF is overexpressed in a variety of human cancers and correlated with poor prognosis in patients of HCC [4], breast cancer [5], non-small cell lung cancer [6], colorectal cancer [7], gastrointestinal stromal tumours [8], pancreatic cancer [9], glioblastoma [10] and oral cancer [11]. Recently, HDGF overexpression has also been delineated to contribute to liver fibrosis [12], epithelial-mesenchymal transition and metastasis [5, 13]. However, the receptor(s) and signaling pathway for HDGF remain elusive. The present study characterized surface nucleolin (NCL) as a receptor for HDGF and evaluated the prognostic and therapeutic potential of NCL for HCC.

## RESULTS

### NCL is an HDGF-binding membrane protein in hepatoma cells

**Figure 1:**
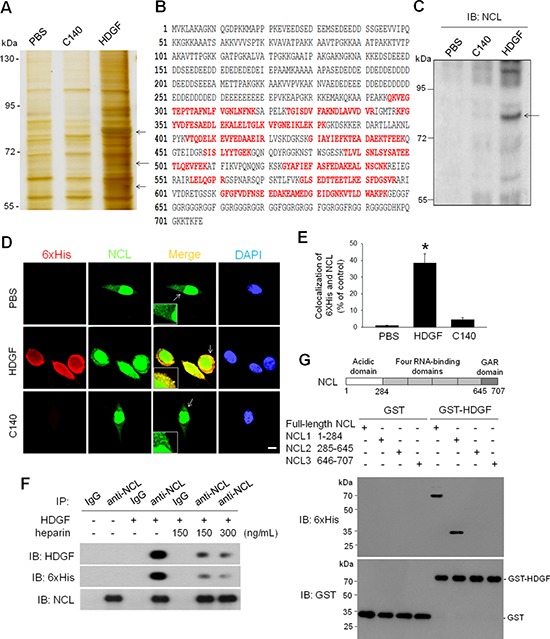
Characterization of NCL as a HDGF-binding membrane protein **A.** Silver staining analysis of the eluted proteins from different affinity columns after separation by 10% SDS-PAGE. The protein bands of interest (asterisk) were digested and identified by LC-MS/MS spectra and NCBI database. **B.** NCL as a HDGF-binding membrane protein. Peptide identification revealed several peptides sequences in red matched in NCL protein. **C.** Immunoblot analysis of the eluted proteins from different affinity columns using an anti-NCL antibody. The arrow indicated the presence of NCL. **D.** Co-localization of exogenous HDGF with NCL in SK-Hep-1 cells by immunofluorescence analysis. After treatment with exogenous HDGF and C140 (10 ng/mL) for 4 hours, cells were permeabilized by Triton X-100 and stained with anti-6xHis (red) and anti-NCL antibodies (green). Bar, 20 μm. **E.** Quantification of co-localization of 6xHis and NCL immunostaining. Data were analyzed using WCIF-Image J software and presented as mean ± SD percentages of the control. *P < 0.05 versus control. **F.** Competition of HDGF binding to NCL by heparin. Membrane proteins of SK-Hep-1 cells were incubated with recombinant HDGF (500 ng/mL) and heparin (150 and 300 ng/mL) for 4 hours. The complex was immunoprecipitated with an anti-NCL antibody and immunoblotted with various antibodies. **G.** GST pull down assay. GST-fused HDGF was added to 6xHis-tagged NCL residues 1–707, residues 1–284, residues 285–645, or residues 646–707 bound to glutathione-Sepharose beads. Proteins on the beads were immunoblotted with anti-6xHis and anti-GST antibodies.

To search for the plausible HDGF receptor(s) in HCC cells, affinity column chromatography and mass spectrum-based proteomic approaches were employed to identify the HDGF-interacting proteins from the membrane fraction of hepatoma SK-Hep-1 cells, which was validated by immunoblot analysis (data not shown). After affinity column chromatography, silver staining analysis revealed several distinct protein bands (from 55–95 kDa) present only in elutes from HDGF, but not C140, affinity column (Figure 1A). By using liquid chromatography tandem mass spectrometry analysis, peptide identification of these excised bands showed that one of the major protein band at 77 kDa was characterized as nucleolin (NCL) with a total score of 948 and 32% sequence coverage to human NCL in NCBI database (accession number: gi189306; Figure 1B, Supplementary Table 1 and 2). A total of 21 peptide sequences were matched to human NCL sequence. Moreover, immunoblot analysis using an anti-NCL antibody further confirmed the enrichment of NCL in the elutes from HDGF affinity column (Figure 1C). Thus, the proteomic studies suggested that NCL is a HDGF-interacting membrane protein in hepatoma cells.

### HDGF interacts with surface NCL via heparin-binding HATH domain

To confirm the interaction of HDGF with surface NCL, immunofluorescence analysis was used to investigate the NCL distribution after exposure to various recombinant HDGF proteins. It was found that exogenous HDGF supply was co-localized with NCL in cytoplasm/plasma membrane of hepatoma cells (Figure 1D–1E). In contrast, exogenous C140 supply exhibited no significant NCL co-localization. To further validate whether such interaction indeed took place in membrane, we used a membrane-labeling carbocyanine dye, Dil, in immunofluorescent analysis [14, 15]. It was observed that DiI staining was co-localized with more than 80% of 6xHis-tagged HDGF immunostaining at surface of hepatoma cells (Supplementary Figure 1A). Similarly, about 10% of NCL immunostaining was co-localized with Dil staining in HDGF-treated cells (Supplementary Figure 1B). These results indicate HDGF binds to NCL in plasma membrane.

Because the heparin-binding HATH domain of HDGF is responsible for the cell surface binding [16], we investigated the influence of excessive heparin on the interaction between HDGF and NCL by co-IP assay. Heparin supply dose-dependently attenuated the binding between HDGF and NCL without affecting the NCL level (Figure 1F). To dissect the HDGF-binding domain within NCL, recombinant NCL proteins encompassing the N-terminal domain (residues 1–284), the central domain (residues 285–645), and the C-terminal argnine-glycine-glycine domain (residues 646–707) were generated for GST pull down assay. The N-terminal domain of NCL was responsible for the interaction between NCL and HDGF (Figure 1G). Together, these results indicate that HDGF directly interacts with cell surface NCL through its HATH domain.

### Exogenous HDGF promotes the translocation and enhances stability of NCL in plasma membrane of hepatoma cells

**Figure 2:**
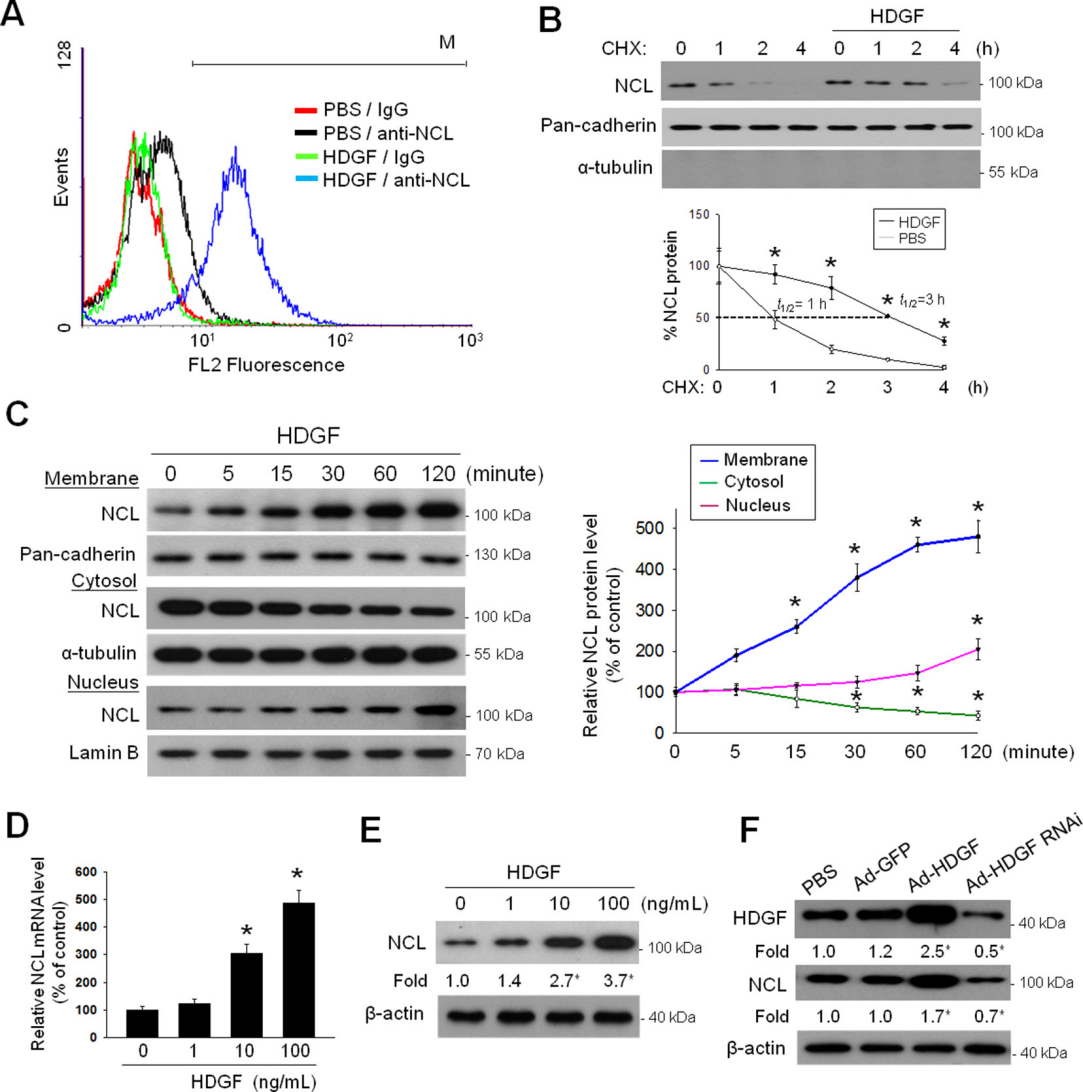
Effect of exogenous HDGF on the membrane translocation and the protein stability of NCL in hepatoma cells **A.** Flow cytometry analysis of cell surface NCL expression after HDGF treatment. After treatment with HDGF (10 ng/mL) for 24 hours, cells were fixed in paraformaldehyde solution. Plasma membrane NCL was stained with an anti-NCL antibody and evaluated by flow cytometry analysis. **B.** Effect of HDGF on NCL stability in plasma membrane. Cells were simultaneously treated with HDGF (10 ng/mL) and cycloheximide (CHX, 50 μg/mL) for 1–4 hours. The protein stability of the purified membrane NCL (from 2 × 10^7^ cells) was monitored by immunoblot analysis. **C.** Time-dependent effect of HDGF on NCL distribution in various subcellular fractions in SK-Hep-1 cells. Plasma membrane, cytosol and nuclear NCL were immunoblotted using an anti-NCL antibody. **D.** Dose effect of HDGF on NCL mRNA expression in SK-Hep-1 cells. After treatment with HDGF for 24 hours, total RNA was isolated for quantitative RT-PCR analysis of NCL mRNA level. **E.** Dose effect of HDGF on NCL protein expression in SK-Hep-1 cells. After treatment with HDGF for 24 hours, protein extracts were isolated for immunoblot analysis of NCL protein level. **F.** Effect of HDGF modulation on NCL protein level by immunoblot analysis. After infection with adenovirus vectors at an MOI of 200 for 72 h, cells were harvested for detecting the protein expression of HDGF and NCL. The data are presented as the mean ± SD as percentages of the control. *P < 0.05 versus control.

Although known as an abundant nuclear protein, NCL shuttles among various subcellular compartments from nucleus, cytoplasm and plasma membrane [17]. To investigate whether HDGF regulated the distribution and expression of NCL, flow cytometry analysis was performed to evaluate the cell surface NCL expression in HDGF-treated SK-Hep-1 cells. HDGF treatment increased the cell surface NCL level in SK-Hep-1 cells (Figure 2A). Subsequently, a cycloheximide (CHX)-chase experiment was performed to determine the stability of membrane NCL. It was found that exogenous HDGF supply significantly extended the half-life of membrane NCL from 1 hour to 3 hours (Figure 2B). By using various subcellular fractions, the time-series studies indicated that HDGF elicited the membrane translocalization of NCL from cytoplasm to plasma membrane in as early as 15 minutes (Figure 2C). To investigate whether HDGF directly regulates NCL expression, quantitative RT-PCR and immunoblot analysis revealed that HDGF dose-dependently increased NCL mRNA and protein levels in SK-Hep-1 cells (Figure 2D–2E). Moreover, ectopic HDGF overexpression by infection with adenovirus vectors encoding HDGF (Ad-HDGF) significantly increased the NCL protein level, whereas HDGF silencing by infection with adenovirus vectors encoding HDGF small interfering RNA (Ad-HDGF RNAi) decreased the NCL protein level in SK-Hep-1 cells (Figure 2F). Therefore, HDGF promotes the translocation and stability of surface NCL during early exposure and ultimately induces NCL upregulation in hepatoma cells after longer treatment.

**Figure 3:**
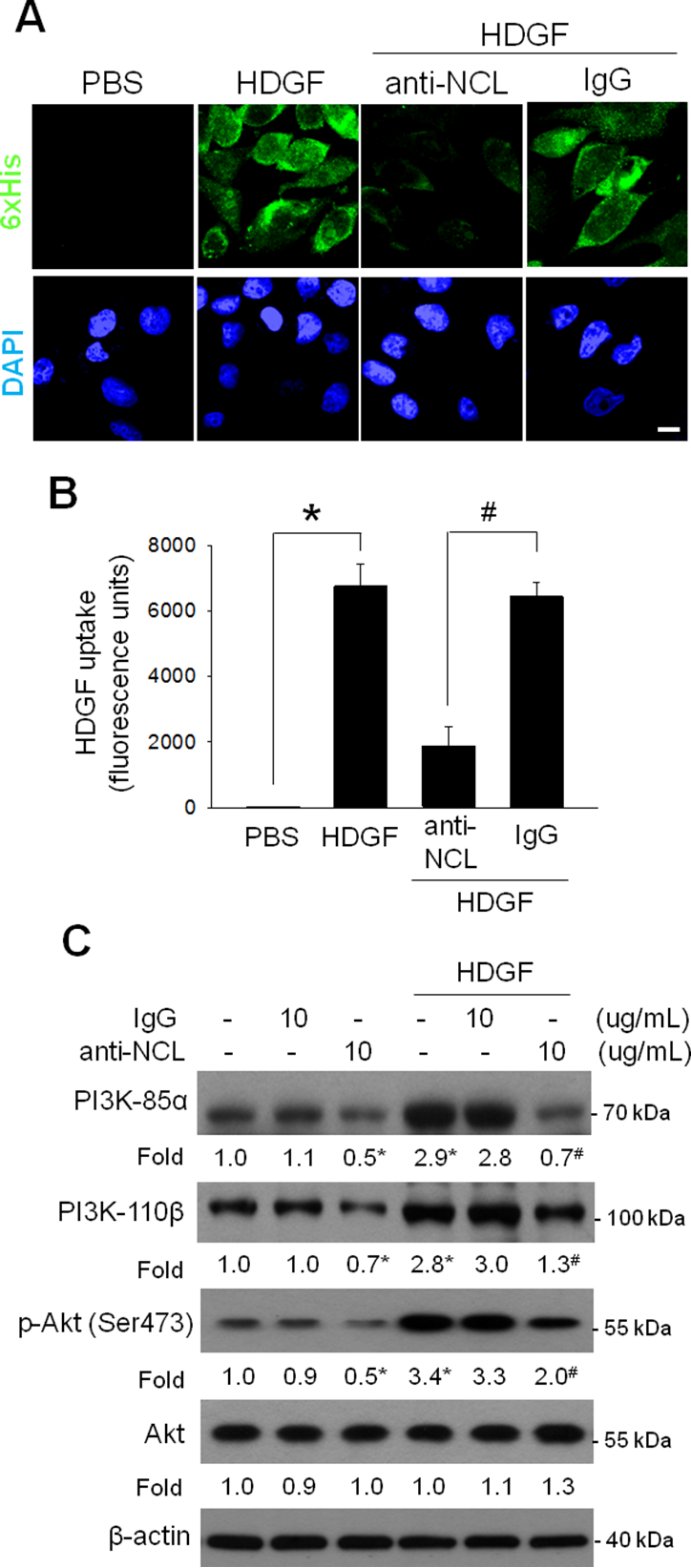
Effect of NCL blockade on HDGF uptake and HDGF-stimulated PI3K/Akt signaling of hepatoma cells **A.** Effect of NCL blockade on HDGF uptake in SK-Hep-1 cells. After treatment with HDGF (10 ng/mL) in the absence or presence of anti-NCL neutralizing antibodies (10 μg/mL) for 4 hours, HDGF staining of cells permeabilized by Triton X-100 was detected using an anti-6xHis antibody and examined by a confocal microscope. Bar, 20 μm. **B.** Effect of NCL blockade on HDGF uptake in SK-Hep-1 cells. After treatment with Alexa Fluor 488 dye–labeled HDGF (100 ng/mL) in the absence or presence of anti-NCL neutralizing antibodies (10 μg/mL) for 4 hours, the amount of fluorescently-labeled HDGF was examined by a spectrofluorometer. *P < 0.05. ^#^*P* < 0.05. **C.** Effect of NCL blockade on HDGF-stimulated PI3K/Akt signaling of SK-Hep-1 cells. After treatment with HDGF (10 ng/ml) for 24 hours, the protein levels of PI3K-85α, PI3K-110β, p-Akt and Akt were assessed by immunoblot analysis. The data are presented as the mean ± SD as percentages of the control. *P < 0.05 versus control. ^#^*P* < 0.05 versus 10 ng/mL HDGF.

To further confirm the function of NCL as a cell surface receptor for HDGF, we examined whether neutralization of surface NCL influenced the HDGF uptake of hepatoma cells. By using immunofluorescence analysis, it was shown that application of anti-NCL neutralizing antibodies prominently reduced intake of HDGF in hepatoma cells (Figure 3A). Moreover, by using fluorescent-labeling HDGF, the cellular uptake assay revealed that prior NCL blockage significantly depleted the fluorescence in HDGF-treated hepatoma cells (Figure 3B). Because HDGF induces the activation of phosphatidylinositol 3-kinase (PI3K)/Akt pathway [18], we examined the effect of NCL neutralization on HDGF-stimulated PI3K/Akt signaling by immunoblot analysis. It was shown that application of anti-NCL antibodies, but not control IgG, abrogated the HDGF-induced PI3K/Akt phosphorylation in hepatoma cells (Figure 3C). These results reveal that surface NCL is involved in the cellular HDGF uptake as well as HDGF-stimulated PI3K/Akt signaling in hepatoma cells.

### Elevated NCL expression is correlated with HCC progression in human HCC tissues

**Figure 4:**
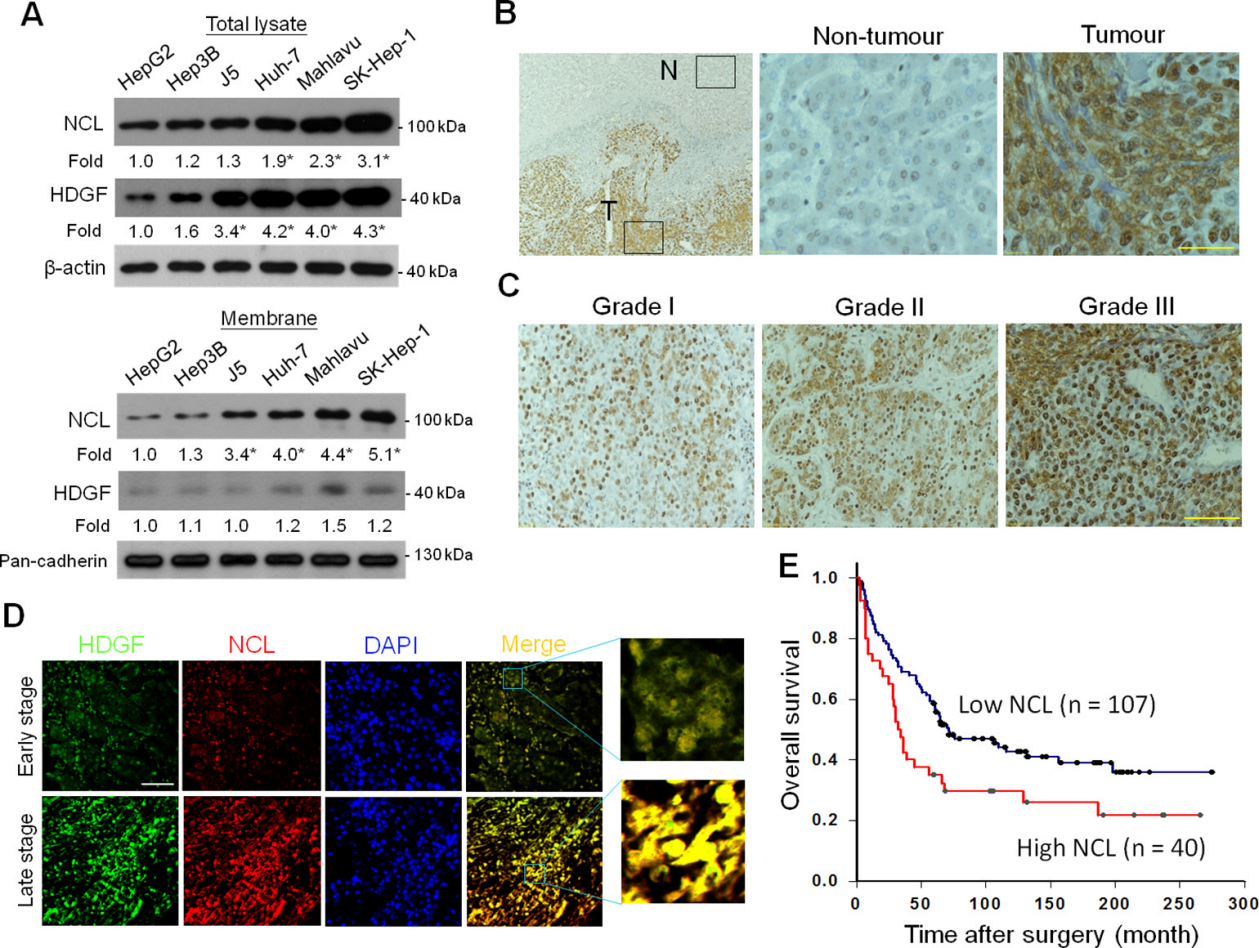
Expression of NCL in human HCC tissues and its association with tumour grade and the survival outcome **A.** NCL and HDGF expression in human hepatoma cell lines. (Upper panel) Total extracts and (Lower panel) membrane extracts were isolated for immunoblot analyses of NCL and HDGF protein levels. The data are presented as the mean ± SD as percentages of the control. *P < 0.05 versus HepG2 cells. **B.** NCL expression in human HCC tissues. (Left) 40 × magnification; (Middle) Non-tumour tissues. 400 × magnification. (Right) Tumour tissues. 400 × magnification. Bar, 100 μm. **C.** NCL expression in different grades of HCC tissues. 200 × magnification. Bar, 200 μm. **D.** Immunofluorescence staining of HDGF (green) and NCL (red) in the different stages of human HCC tissues. Bar, 200 μm. **E.** Kaplan-Meier analysis of the overall survival for HCC patients with different NCL expression levels.

HDGF overexpression is correlated with tumor progression and poor survival outcome for HCC patients [4]. However, the prognostic role of NCL has never been studied in HCC. Thus, we evaluated whether NCL overexpression is associated with tumour malignancy in HCC. By using a panel of hepatoma cells with different differentiation status, immunoblot analysis showed that the cellular NCL level exhibited an escalated trend from well-differentiated (HepG2 and Hep3B cells), intermediate-differentiated (J5 and Huh-7 cells), to poorly differentiated (Mahlavu and SK-Hep-1) hepatoma cells (Figure 4A). Interestingly, such trend of NCL upregulation was even more pronounced when analyzing the membrane NCL level among these hepatoma cells. Thus, these results implicated that NCL overexpression may be associated with loss of differentiation features in hepatoma cells. Subsequently, immunohistochemial analysis of NCL expression was performed using resected HCC samples (*n* = 147) to delineate the role of NCL in HCC progression. Using the scoring system for immunostaining intensities, the statistical analysis demonstrated that the NCL labeling index (LI) in the tumour tissues were higher than that in the non-tumour tissues (*P* < 0.001; Figure 4B). Besides, the NCL LI increased as the tumour grades proceeded (Figure 4C). To evaluate the clinical relevance of HDGF and NCL expression in HCC, we examined the expression patterns of HDGF and NCL in the surgical specimens of the early-stage HCC (TNM stage I or II) and the late-stage HCC (TNM stage III or IV). Immunofluorescence analysis revealed that the co-localization of HDGF and NCL in the late-stage HCC specimens was higher than that in the early-stage HCC specimens (Figure 4D). Statistical analyses further revealed the NCL LI was correlated positively with tumour grade (*P* = 0.010), vascular invasion (*P* = 0.034), and alpha-fetoprotein (αFP) level (*P* = 0.006; Table 1). There was a positive correlation between NCL LI and HDGF expression (*P* = 0.028). These results show that NCL overexpression is positively correlated with HCC progression.

**Table 1:** Correlation between NCL and clinicopathologic parameters of HCCs

Variant	Low (*n* = 107)	High (*n* = 40)	*p* value
**Gender**			NS (0.59)^α^
Female (*n* = 30)	23	7	
Male (*n* = 117)	84	33	
**Cirrhosis**			NS (0.056)^α^
With (*n* = 88)	59	29	
Without (*n* = 59)	48	11	
**HBsAg**			NS (0.59)^α^
Positive (*n* = 102)	74	28	
Negative (*n* = 39) (missing 6)	30	9	
**Anti-HCV**			NS (0.78)^α^
Positive (*n* = 32)	23	9	
Negative (*n* = 109) (missing 6)	81	28	
**HCC grades**			^*^0.010^α^
Well (*n* = 35)	32	3	
Moderate (*n* = 71)	50	21	
Poor (*n* = 41)	25	16	
**Capsule**			NS (0.81)^α^
With (*n* = 82)	61	21	
Without (*n* = 65)	46	19	
**Vascular invasion**			^*^0.034^α^
Without (*n* = 73)	59	14	
With (*n* = 74)	48	26	
**Tumour numbers**			NS (0.40)^α^
Solitary (*n* = 110)	82	28	
Multi (≥ 2) (*n* = 37)	25	12	
**Age**	53.8 ± 13.5	55.2 ± 12.8	NS (0.59)^β^
**Tumour size (cm)**	6.87 ± 4.21	5.65 ± 3.14	NS (0.09)^β^
**αFP (ng/mL)**	2712 ± 7191	3404 ± 11329	^*^0.006^γ^

* Statistically significant (*p* < 0.05)

α Chi-Square test

β Student *t* test

γ Mann-Whitney *U* Test.

NS: not significant.

### NCL is a novel prognostic factor for survival of HCC patients

To evaluate the prognostic potential of NCL expression in HCC, the patients were divided into 2 groups based on their NCL labeling intensities for survival analysis. Kaplan–Meier analysis showed that the NCL LI was inversely correlated with the overall survival rate of HCC patients after surgery (*P* = 0.016; Figure 4E). The patients with a strong NCL LI had low survival rates compared with the patients with a weak NCL LI (Figure 4E). However, NCL was not a significant prognostic marker for the disease-free survival of HCC patients (*P* = 0.078).

Univariate analysis in the Cox proportional hazard model revealed that NCL expression, serum αFP levels, tumour capsule, tumour size, vascular invasion and grade were independent variables to predict the overall survival (*P* < 0.05; Table 2). Notably, although not related to disease-free survival, multivariate analysis revealed that the high NCL expression in HCC could predict shorter patient's survival after resection and serve as an independent prognostic factor (*P* < 0.05; Table 2). These results show that NCL provides the prognostic potential for the survival outcome in HCC patients.

**Table 2:** Correlation of clinicopathologic factors and overall survival of HCC patients

	Univariate			Multivariate		
	Risk	95% CI	*P*	Risk	95% CI	*P*
**Biomarkers**						
Nucleolin	1.30	1.04–1.62	0.018	1.32	1.06–1.66	0.013
**Clinical parameters**						
Age	0.90	0.59–1.38	NS	—	—	—
Gender	1.32	0.78–2.23	NS	—	—	—
αFP	1.68	1.11–2.55	0.013	—	—	NS (0.11)
HBV	1.36	0.84–2.20	NS	—	—	—
HCV	0.79	0.48–1.30	NS	—	—	—
Viremia (B or C)	2.87	1.13–4.89	0.021	—	—	NS (0.35)
Cirrhosis	1.53	1.00–2.36	0.050	—	—	NS (0.28)
**Pathologic parameters**						
Tumour capsule	0.46	0.30–0.70	< 0.001	0.52	0.33–0.83	0.006
Tumour size	1.95	1.28–2.95	0.002	—	—	NS (0.54)
Tumour number	1.34	0.85–2.12	NS	—	—	—
Vascular invasion	3.68	2.38–5.69	< 0.001	2.72	1.71–4.32	< 0.001
Grade	1.56	1.17–2.08	0.002	—	—	NS (0.16)

Nucleolin, low or high; Age, ≥ 60 or < 60 years; Gender, male or female; serum α-FP, ≥ 400 or < 400; HBV+, with or without; HCV+, with or without; Cirrhosis, with or without; Tumour capsulation, with or without; Tumour size, ≥ 5 or < 5 cm; Tumour number, solitary or ≥ 2; Grades, I + II or III + IV; Pathologic stages, I + II or III + IV. NS: not significant.

### NCL overexpression elicits HDGF upregulation and promotes the oncogenic behaviours via PI3K/Akt siganling in hepatoma cells

**Figure 5:**
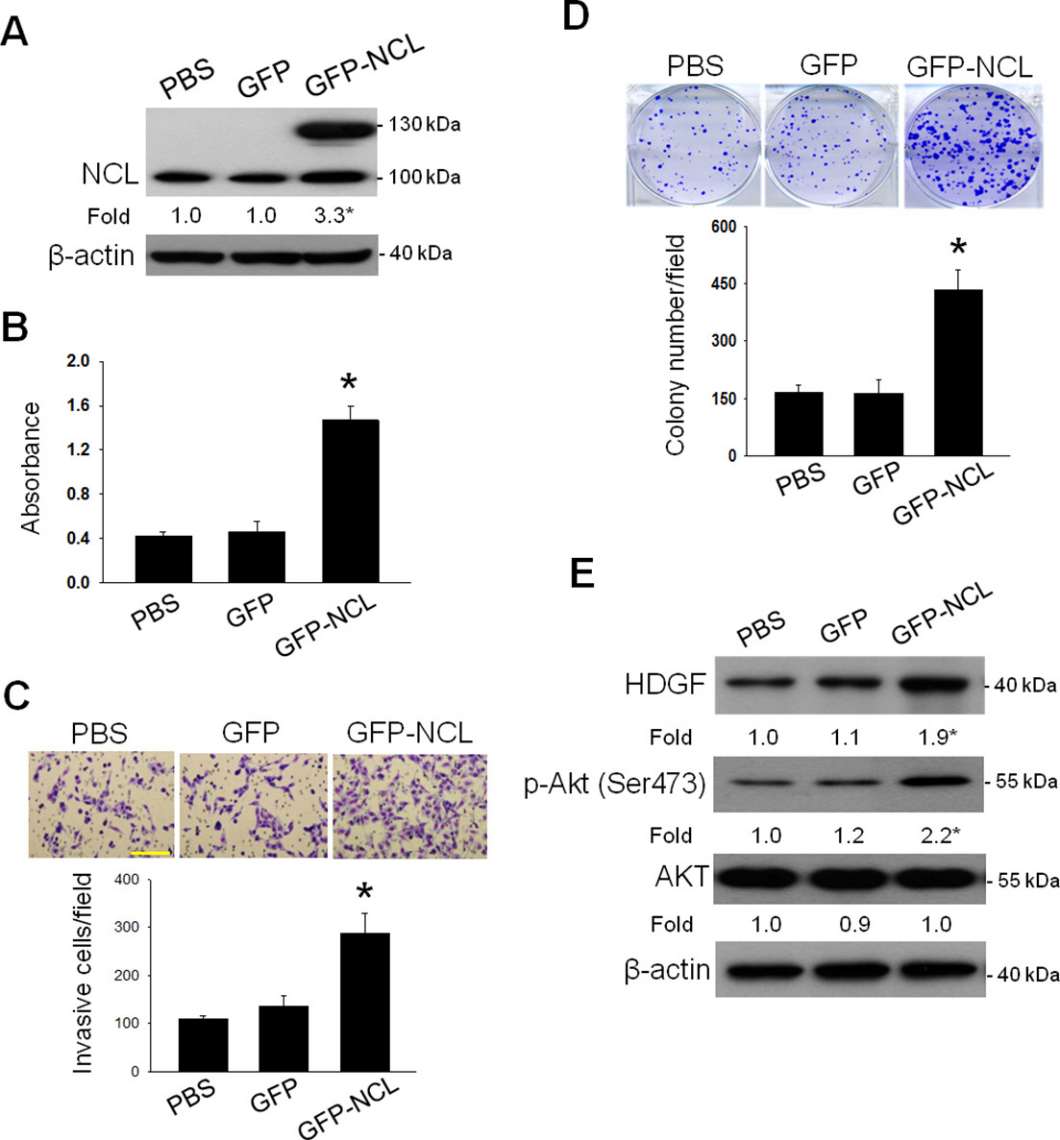
Influence of NCL overexpression on the oncogenic behaviors and HDGF/Akt signaling in hepatoma cells After transfection with pEGFP-C1 vector encoding NCL fused with green fluorescent protein (NCL-GFP) for 48 hours, cells were harvested for subsequent analysis. **A.** Effect of NCL-GFP transfection on NCL expression in SK-Hep-1 cells. The protein levels of NCL were assessed by immunoblot assay. **B.** Effect of NCL-GFP transfection on proliferation of SK-Hep-1 cells. Cell proliferation was assessed by MTT assay. **C.** Effect of NCL-GFP transfection on invasion of SK-Hep-1 cells. The invasion capability was assessed by Boyden chamber assay. Bar, 250 μm. **D.** Effect of NCL-GFP transfection on anchorage-independent growth of SK-Hep-1 cells. The colony-forming capability was determined by crystal violet and counted. **E.** Effect of NCL overexpression on the protein expressions of HDGF and p-Akt in SK-Hep-1 cells. The protein levels of HDGF, p-AKT and AKT were assessed by immunoblot assay. The data are presented as the mean ± SD as percentages of the control. *P < 0.05 versus control.

We then investigated whether the cellular NCL level affected the oncogenic behaviours of hepatoma cells. Ectopic NCL overexpression using expression vector encoding NCL fused with green fluorescent protein (NCL-GFP) increased the cellular NCL protein level in SK-Hep-1 cells by approximately 3.3-fold over the control (Figure 5A). Moreover, the NCL-transfected hepatoma cells exhibited significantly enhanced tumorigenicity of hepatoma cells including proliferation, invasiveness and anchorage-independent growth (Figure 5B–5D). Since PI3K/Akt is the downstream effector of HDGF, we investigated whether NCL overexpression influenced the HDGF/Akt signaling in hepatoma cells. Immunoblot analysis showed that NCL overexpression significantly increased the HDGF protein level and stimulated the Akt phosphorylation in hepatoma cells (Figure 5E). Thus, NCL overexpression elicits HDGF upregulation and promotes the malignancy of hepatoma cells. Similarly, NCL overexpression also enhanced the oncogenic behaviors in another hepatoma HepG2 cells (Supplementary Figure 2).

**Figure 6:**
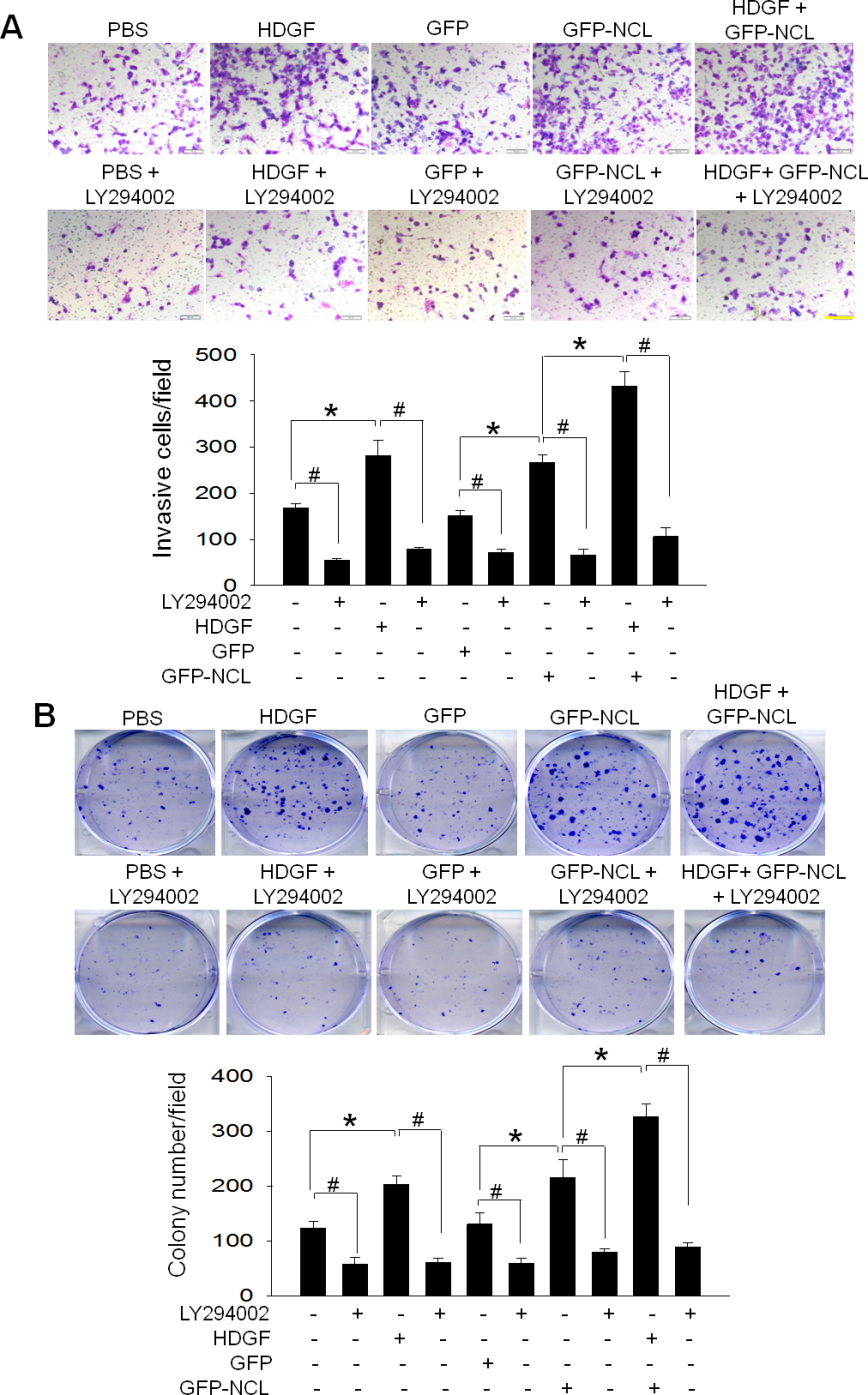
Effect of LY294002 on HDGF/NCL-stimulated the oncogenic behaviors in hepatoma cells After treatment with HDGF (10 ng/mL) or/and transfection with pEGFP-C1 vector encoding NCL in the absence or presence of LY294002 (10 μM) for the indicated times, SK-Hep-1 cells were harvested for subsequent analysis. **A.** Effect of LY294002 on HDGF/NCL-stimulated the invasion of SK-Hep-1 cells. The invasion capability was assessed by Boyden chamber assay. Bar, 250 μm. **B.** Effect of LY294002 on HDGF/NCL-stimulated the anchorage-independent growth of SK-Hep-1 cells. The colony-forming capability was determined by crystal violet and counted. The data are presented as the mean ± SD as percentages of the control. *P < 0.05. ^#^*P* < 0.05.

To confirm the role of PI3K/Akt signaling in HDGF/NCL-induced tumorigenicity, a pharmaceutical PI3K inhibitor, LY2940002, was used. It was found LY2940002 application significantly suppressed the HDGF- or NCL-stimulated invasion and colonies formation in hepatoma cells (Figure 6A–6B). Above all, LY2940002 treatment potently abolished the compounding effect of HDGF supply and NCL overexpression on oncogenic behaviors of hepatoma cells.

### NCL knockdown attenuates the basal and HDGF-stimulated oncogenic behaviours and PI3K/Akt pathway in hepatoma cells

**Figure 7:**
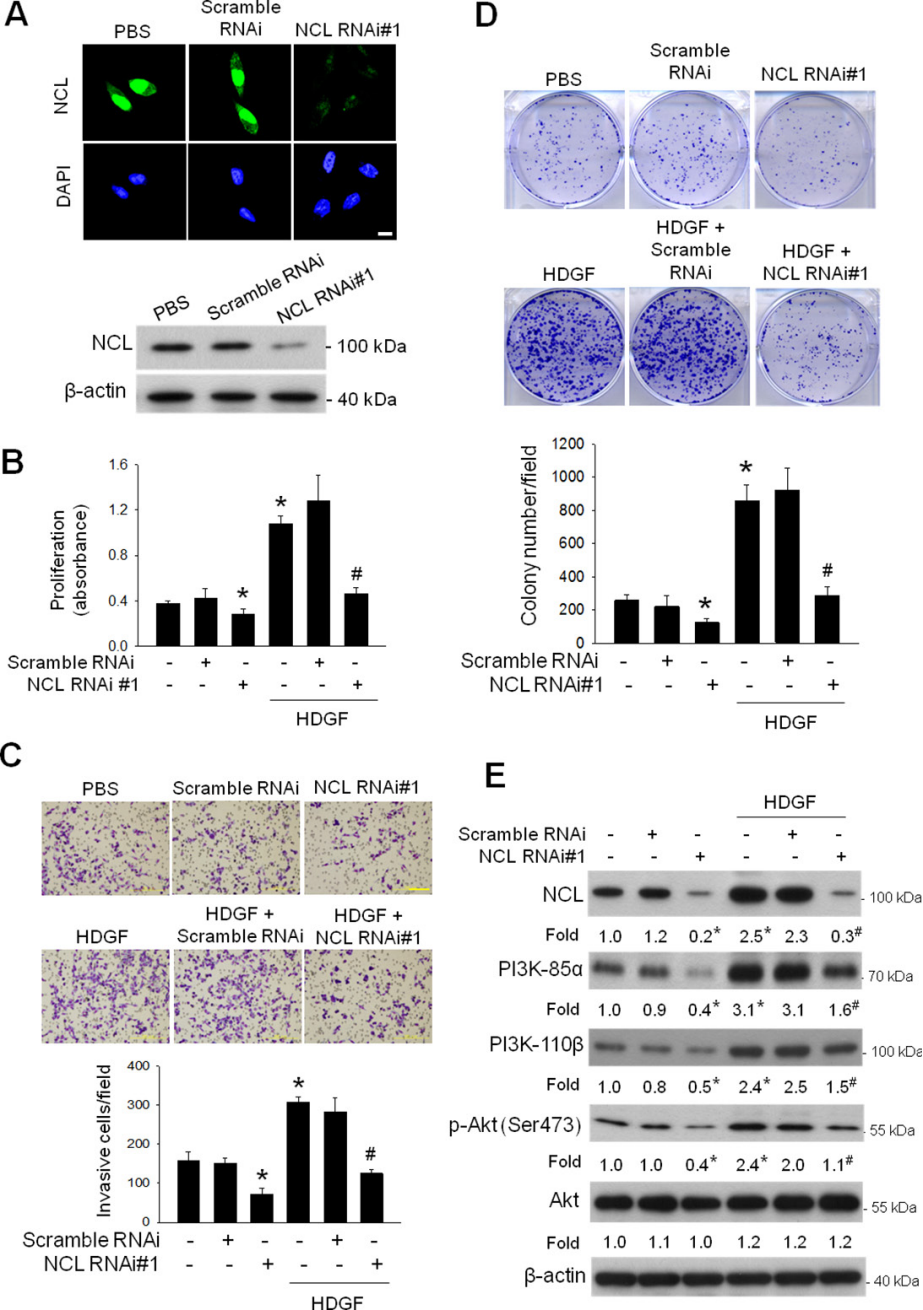
Effect of NCL knockdown on the basal and HDGF-stimulated oncogenic behaviours and PI3K/Akt signaling of hepatoma cells After treatment with HDGF (10 ng/mL) in the absence or presence of NCL siRNA#1 (0.5 μg) for the indicated times, cells were harvested for the subsequent analysis. **A.** Effect of NCL siRNA#1 on NCL protein level in SK-Hep-1 cells. After transfection for 72 hours, NCL expression was detected by (Upper panel) immunofluorescence assay and (Lower panel) immunoblot analysis using an anti-NCL antibody. Bar, 20 μm. **B.** Effect of NCL siRNA#1 on proliferation of SK-Hep-1 cells. After treatment with HDGF for 24 hours, cell proliferation was assessed by MTT assay. **C.** Effect of NCL siRNA#1 on invasiveness of SK-Hep-1 cells. After treatment with HDGF for 24 hours, the invasion capability was assessed by Boyden chamber assay. Bar, 250 μm. **D.** Effect of NCL siRNA#1 on anchorage-independent growth of SK-Hep-1 cells. After treatment with HDGF for 10 days, the colony-forming capability was detected by crystal violet solution and counted. **E.** Effect of NCL siRNA#1 on PI3K/Akt signaling of SK-Hep-1 cells. After treatment with HDGF for 24 hours, the protein levels of NCL, PI3K-85α, PI3K-110β, p-Akt and Akt were assessed by immunoblot assay. The data are presented as the mean ± SD as percentages of the control. *P < 0.05 versus control. ^#^*P* < 0.05 versus 10 ng/mL HDGF.

**Figure 8:**
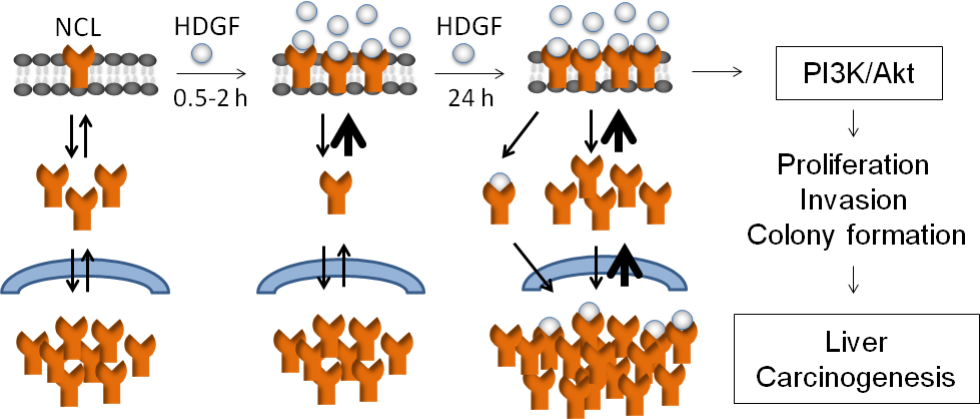
Hypothetical model for HDGF-induced NCL membrane trafficking from cytoplasm (in 0.5–2 hours), NCL upregulation (in 24 hours), activation of PI3K/Akt pathway and oncogenic behaviours of hepatoma cells, which ultimately contributes to liver carcinogenesis

Subsequently, we evaluated the effect of NCL knockdown on the oncogenic behaviours of hepatoma cells using small interfering RNA (siRNA). Immunofluorescence and immunoblot analyses validated the efficacy of NCL siRNA in depleting NCL level in hepatoma cells (Figure 7A). Moreover, NCL knockdown significantly attenuated the basal and HDGF-stimulated proliferation, invasiveness and anchorage-independent growth of hepatoma cells (Figure 7B–7D). Immunoblot analysis further showed that NCL knockdown potently inhibited the basal and HDGF-stimulated PI3K expression and Akt phosphorylation in hepatoma cells (Figure 7E). Similar results were observed using another hepatoma Huh-7 cells (data not shown), or another NCL RNAi (Supplementary Figure 3). Together, these findings indicate NCL knockdown attenuates the basal and HDGF-stimulated oncogenic behaviours and PI3K/Akt pathway in hepatoma cells. In summary, we herewith proposed the hypothetical model for HDGF-induced NCL membrane targeting and accumulation, which activates PI3K/Akt signaling to promote liver carcinogenesis (Figure 8).

## DISCUSSION

The present study employed various approaches including affinity chromatography, LC-MASS peptide identification, immunofluorescence analysis, co-IP and GST pull down assays to demonstrate HDGF directly binds to surface NCL and regulates the NCL membrane translocalization, NCL gene expression, NCL/PI3K/Akt pathway and oncogenic behaviours in hepatoma cells. The histological studies using human HCC samples unveil the positive correlation of NCL expression with tumour grade, vascular invasion and HDGF expression in HCC patients. Moreover, NCL overexpression constitutes an independent prognostic factor in predicting the overall survival of HCC patients. Above all, NCL inactivation suppresses the HDGF-stimulated oncogenic behaviours and PI3K/Akt activities in hepatoma cells, implicating the potential of NCL as a novel therapeutic target for HCC. Collectively, these results provide the compelling evidence supporting NCL participates in liver carcinogenesis through transmitting HDGF signaling.

By demonstrating the binding of HDGF with NCL in plasma membrane, our results are in agreement with a recent study, in which the interaction and co-localization between HDGF and NCL takes place in the ribonucleoprotein complex [19]. Nevertheless, the present study has further delineated that HDGF binds to the N-terminal domain of NCL through its heparin-binding HATH domain. This is also consistent with the previous study that the HATH domain (particularly Lys96 residue) is critical to the cellular uptake of HDGF [16]. By locating in the extracellular space, the N-terminal domain of NCL consists of high content of acidic residues and hence facilitates the binding with ligands rich in basic amino acids, such as midkine and heparan-binding domain of matrix proteins [20, 21]. However, several fundamental issues regarding the interaction between HDGF and NCL remain to be solved. For example, the binding affinity (*i.e.*, the dissociation constant) between NCL and HDGF is not determined yet.

NCL, a multiple-function protein shuttles between the cell surface and the nucleus, is involved in RNA biogenesis, cell proliferation, differentiation, adhesion, mitogenesis and angiogenesis [22, 23]. Interestingly, surface NCL is also a receptor for various molecules including cytokines (like midkine and pleiotrophin) [21, 24], vascular-targeting factors (endostatin and tumour-homing peptide F3) [25, 26] and some glycosaminoglycans [27]. Because both HDGF and NCL bind to heparin, it seems possible that HDGF may interact with NCL in a glycoprotein-dependent manner. However, besides proteoglycan, additional molecules may participate in the formation of surface HDGF-NCL signaling complex because several distinct proteins other than NCL are present in elutes from HDGF affinity column chromatography (data not shown). It is hypothesized that NCL may serve as the high-affinity receptor for HDGF whereas the other co-receptor proteins may confer the specificity for HDGF recognition in the oncogenic HDGF/NCL signaling axis. Studies are currently undergoing to characterize the existence and functions of these HDGF-binding membrane proteins.

The identification of NCL as a receptor for HDGF brings important insights on the signaling pathway of HDGF. NCL has been demonstrated to bind directly to PI3K complex in lymphoma cells during CD21 activation [28]. Thus, HDGF may directly induce Akt activation in hepatoma cells through NCL. Alternatively, it has been reported surface NCL is associated with other oncogenic receptors including ErbB and c-Met [29, 30]. Thus, in addition to direct PI3K/Akt activation, HDGF/NCL axis may also participate in liver carcinogenesis through interaction with other oncogenic pathways. Moreover, our studies unveiled that modulation of NCL expression could regulate HDGF expression in a feedback manner through as yet unidentified mechanism. Thus, the coordinate and reciprocal interaction between HDGF and NCL strongly advocates the important role of HDGF/NCL signaling axis in liver carcinogenesis.

To our knowledge, the present study has presented the first evidence for the prognostic function of NCL that NCL expression is correlated with tumour progression and inversely associated with the survival outcome of HCC patients after surgery. NCL overexpression is observed in several types of cancer with high proliferative rates including melanoma and breast cancer [31, 32]. Recently, the prognostic role of NCL has been reported in pediatric intracranial ependymoma, cutaneous melanocytic lesions and gastric cancer tissues, in which nuclear NCL overexpression is associated with the tumour progression of pediatric ependymoma, melanoma and gastric cancer [33–35]. This is in accordance with the pro-oncogenic function of NCL in our HCC study. Future studies are warranted to delineate whether NCL may serve a novel diagnostic marker for liver diseases such as hepatitis and liver fibrosis/cirrhosis, which proceed prior to the development of HCC.

Recent evidence advocates that surface NCL may facilitate as a novel molecular target for cancer therapy. At present, there are several anti-neoplastic agents targeting at cell surface NCL including AS1411, an nucleic acid-based aptamer that binds to cell surface NCL [36], and HB-19 pseudopeptide, which acts directly on the C-terminal domain of NCL [22]. By unveiling NCL serves as a HDGF receptor, the present study echoes such view that surface NCL may constitute a therapeutic target for liver cancer. Our study has further demonstrated the anti-tumor efficacy of NCL inactivation via knockdown and antibodies neutralization in suppressing the tumorigenicity of hepatoma cells even in the presence of excessive HDGF. Further studies using the pre-clinical HCC animal models are required to fully optimize and appreciate the therapeutic potential of NCL-targeting modalities for HCC.

In conclusion, this study provides the first evidence that NCL is a HDGF receptor and mediates the HDGF-stimulated oncogenic behaviours and PI3K/Akt pathway. NCL expression is positively correlated with tumour grade, vascular invasion and shorter survival outcome in HCC patients. Therefore, HDGF/NCL axis participates in liver carcinogenesis and facilitates a novel diagnostic and therapeutic target for HCC.

## MATERIALS AND METHODS

### Cell cultures

HepG2 and Hep3B cells were cultured in minimum essential medium (MEM), Mahlavu and SK-Hep-1 cells were cultured in Dulbecco's modified Eagle's medium (DMEM). These four cell lines were from the American Type Culture Collection (Manassas, VA). J5 and Huh-7 were cultured in DMEM and purchased from the Food Industry Research and Development Institute (Hsinchu, Taiwan). Both MEM and DMEM supplemented with 10% fetal calf serum (FCS), 100 IU/mL penicillin, 100 μg/mL streptomycin, and 2 mM L-glutamine in 5% CO_2_ at 37°C.

### Isolation of HDGF-binding membrane proteins

SK-Hep-1 cells were washed twice with cold PBS and collected with EDTA. After centrifugation, the pellet was re-suspended in 1 mL ice-cold hypotonic buffer (10 mM HEPES pH 7.9, 0.5 mM dithiothreitol, protease inhibitors: 0.5 mM phenylmethylsulfonyl fluoride, aprotinin, pepstatin, leupeptin (10 μg/ml each); and phosphatase inhibitors: 50 mM NaF, 30 mM β-glycerophosphate, 1 mM Na_3_VO_4_, and 20 mM ρ-nitrophenyl phosphate). The cells were disrupted with a tight-fitting Dounce homogenizer. The homogenate was checked under Olympus phase contrast microscope and no intact cell could be observed. The homogenate was centrifuged to remove the nuclei and mitochondria at 8000 g for 10 minutes. The supernatant was centrifuged at 100000 g for 30 minutes. The membrane fraction, obtained as the pellet, was dissolved in 200 μL ice-cold hypotonic buffer containing 1% Triton X-100 for 1 hour.

### Identification of HDGF-binding membrane proteins

The affinity columns were prepared by incubation of recombinant C140 or HDGF (5 mg) with 2 mL Ni-NTA agarose (50% in PBS; Qiagen, Hilden, Germany). The membrane proteins (5 mg) were incubated with the affinity columns for 12 hours at 4°C, and unbound proteins were removed by washing with phosphate buffered saline (PBS). The fractions were collected by elute buffers (20 mM Tris-HCl, pH 7.9, 500 mM NaCl, 200 mM imidazole). The elutes from various affinity columns were separated by electrophoresis on 10% SDS-polyacrylamide gel and subjected to silver staining. The silver-stained gel pieces were excised for in-gel digestion. The remaining peptides from the gel piece were further analyzed by Nano-HPLC-MS/MS (Thermo Finnigan, San Jose, CA). The resulting MS/MS spectra were searched against NCBI database using Mascot search engine (http://www.matrixscience.com, Matrix Science Ltd., UK). Proteins identified with a *P* value less than 0.05 were considered candidate hits. Information of the identified proteins was referred to the Human Protein Reference Database (http://www.hprd.org).

### HDGF proteins, HDGF antibodies, adenovirus vectors, and other reagents

Recombinant HDGF proteins and anti-HDGF antibodies were generated as previously described [4]. The human HDGF cDNA was amplified from a human fetal brain cDNA library (Stratagene, La Jolla, CA) using the polymerase chain reaction (PCR). The PCR primers used to clone the human HDGF cDNA were designed based on the HDGF sequence in the GenBank database. All HDGF fragments were amplified by PCR using primers as follows: HDGF, 5′-CCGCATATGTCGCGATCCAACC GGCAGAAG-3′ (forward) and 5′-CGCGGATCCCTACAGGCTCTCATGATC-3′ (reverse); C140, 5′-CGCCATATGCAGTCCTCCCAGAAAAAG-3′ (forward) and 5′-CGCGGATCCCTACAGGCTCTCATGATC-3′ (reverse). After the DNA sequencing analysis, the PCR-amplified cDNA of all HDGF fragments was subcloned into the NdeI and BamHI sites of the pET15b vector (Novagen, Madison, WI) and transformed into BL21 cells (DE3, pLysS; Novagen). All 6xHistidine-tagged recombinant proteins were purified on an NTA-agarose affinity column (Qiagen, Hilden, Germany), desalted on a G25 Sephadex column (Amersham Pharmacia, Little Chalfont, United Kingdom), and passed through a Detoxi-Gel column (Pierce Biotechnology, Rockford, IL) to minimize contamination by endotoxins. Purity and correct molecular weight of the recombinant proteins were examined by coomassie blue staining of SDS-PAGE gels and immunoblot analysis using an anti-6xHis antibody (Santa Cruz, CA). Rabbit anti-HDGF pAbs were generated by the periodic injection of recombinant HDGF into rabbits. The serum was collected from immunized animals and analyzed using immunoblot analysis.

The recombinant adenoviruses containing green fluorescent protein (Ad-GFP), HDGF cDNA (Ad-HDGF), and HDGF small-interfering RNA (Ad-HDGF RNAi) were generated as previously described [12].

FCS, DMEM, MEM and 1,1′-dioctadecyl-3,3,3′, 3′-tetramethylindocarbocyanine (DiI) dye were obtained from Invitrogen Corp. (Carlsbad, CA). Other chemicals were obtained from Sigma-Aldrich Co. (St. Louis, MO).

### Co-immunoprecipitation assay

Co-immunoprecipitation assay was performed as previously described [37, 38]. The membrane proteins (500 μg/mL) were incubated with HDGF protein (500 ng/mL) for 4 hours at 4°C, and then immunoprecipitated using an anti-NCL antibody (Sigma-Aldrich) for 1 hour at 4°C. Immunocomplexes were collected on 15 μL protein A/G beads (Calbiochem, San Diego, CA). The precipitated proteins were washed three times with 1% NP-40 lysis buffer (20 mM Tris-HCl, 150 mM NaCl, 1 mM EDTA, 1 mM dithiothreitol, 1% Nonidet P-40, 10% glycerol, 1 mM sodium orthovanadate, and one protease inhibitor mixture tablet), boiled for 5 minutes in SDS sample buffer, and subjected to immunoblot analysis with anti-HDGF, anti-6xHis and anti-NCL antibodies.

### GST pull down assay

GST pull down assay was assessed as previously described [39]. The nucleolin (NCL) constructs including NCL1 (residues 1–284), NCL2 (residues 285–645), and NCL3 (residues 646–707) were generated from a human NCL cDNA clone [21, 40] and subcloned into the NdeI and BamHI sites of the pET15b vector (Novagen, Madison, WI) and transformed into BL21 cells (DE3, pLysS; Novagen). All recombinant NCL proteins were generated and purified by means of their N-terminal 6xHistidine tag.

The expression plasmid pGST-HDGF vector was constructed for production of GST-fused HDGF. After transformation, *E. coli* BL21(DE3) cells carrying GST expression vectors were cultured in 3 mL of LB media at 37°C to the mid-log phase. Isopropylthio-β-D-galactoside (IPTG) was then added to a final concentration of 1 mM to induce the expression of GST fusion proteins. After culturing for 3 hours, BL21 cells were pelleted by centrifugation and suspended in 100 μL of a lysis buffer, B-Per (Pierce), containing 10 μL of leupeptin, aprotinin, and 4-(2-aminoethyl)-benzenesulfonyl fluoride. The suspension was centrifuged again at 10000 rpm for 5 minutes at 4°C. Glutathione-Sepharose 4B beads (20 μL) (GE Healthcare, Piscataway, NJ) were added to the supernatant and the mixture was incubated under shaking for 1 hour at 4°C. The beads were washed 3 times with NETN buffer (20 mM Tris-HCl, pH 8.0, 100 mM NaCl, 1 mM EDTA, 0.5% NonidetP-40). After washing, the beads were added to the lysate (300 μL) prepared from *E. coli* lysate containing 6xHistidine-tagged NCL proteins. The reaction mixture was incubated on ice for 1 hour to allow binding between GST-HDGF proteins and 6xHistidine-tagged NCL proteins. The beads were subsequently washed with NETN buffer. An equal volume of 2x electrophoresis sample buffer was added to the beads, proteins were extracted from the beads by heating at 95°C for 5 minutes. Proteins were finally analyzed by SDS-PAGE and immunoblot analysis.

### Immunofluorescence analysis

Immunofluorescence staining was performed as previously described [17]. Cells were fixed with either 4% paraformaldehyde for monitoring the cell surface NCL staining and 4% paraformaldehyde/0.2% Triton X-100 for monitoring the intracellular NCL staining. Fixed cells were incubated with the antibodies against HDGF (1:1000 dilution), 6xHis (1:100 dilution; Santa Cruz, CA) and NCL (1:1000 dilution; Sigma-Aldrich). The plasma membrane were identified using the well-characterized fluorescent marker DiI. The nuclei were counterstained with 4′,6-diamidino-2-phenylindole (DAPI). Confocal double-immunostaining images were captured with a confocal microscope (ZEISS LSM PASCAL, ZEISS, German). The WCIF-Image J software (National Institutes of Health, Bethesda, MD) was used for measurement and quantification analysis of co-localizing images.

### HDGF uptake assay

6xHistidine-tagged recombinant HDGF was labeled with Alexa Fluor488 reactive dye and purified according to the manufacturer's instructions (Molecular Probes, Invitrogen). The cells were incubated in DMEM supplemented with Alexa Fluor488 dye-labeled HDGF (100 ng/ml) in the absence or presence of an anti-NCL neutralizing antibody (10 μg/mL; Santa Cruz, CA) for 4 hours. Subsequently, SK-Hep-1 cells were washed with PBS and lysised with cell lysis buffer (50 mM Tris, 150 mM NaCl, 1 mM Na-orthovanadae, 1 mM EDTA). The fluorescence of Alexa Fluor488 dye-labeled HDGF was measured using a spectrofluorometer (FLUOstar galaxy, BMG Labtech GmbH, Offenburg, Germany) with excitation set at 485 nm and emission set at 520 nm.

### Immunoblot analysis

Immunoblot analysis was performed as previously described [11]. Ten μg of cell lysate was subjected to electrophoresis on 10% SDS-PAGE. Primary antibodies were used to detect HDGF (1:10000 dilution), NCL (1:10000 dilution; Sigma-Aldrich), PI3K-85α (1:1000 dilution; Santa Cruz), PI3K-110β (1:1000 dilution; Santa Cruz), p-AKT Ser473 (1:1000 dilution; Santa Cruz) and AKT (1:5000 dilution; Santa Cruz).

### Determination of NCL stability

Protein stability assay was performed as previously described [17]. After incubation with HDGF (10 ng/mL) for the indicated times, cells were treated with cycloheximide (50 μg/mL) for the various time intervals and subjected to immunoblot analysis to determine the half-life of NCL protein.

### Flow cytometry analysis of cell surface NCL level

Flow cytometry analysis was performed as previously described [41]. Fixed cells were incubated with either anti-NCL antibodies (10 μg/mL; Santa Cruz) or control-IgG for 1 hour on ice, follow by adding an Alexa Fluor 488-conjugated secondary antibody (1:50 dilution; Molecular Probes, Eugene, Oregon). The antibody-treated cells were washed and re-suspended in 50 μL PBS containing 2 μg/mL propidium iodide to distinguish between live and dead cells, and 10000 cells per sample were analyzed using a FACSCalibur flow cytometer (BD Biosciences, San Jose, CA) equipped with CellQuest software.

### Quantitative reverse transcription-polymerase chain reaction (qRT-PCR)

One-twentieth of the complementary DNA generated was used as a template for real time PCR analysis. Amplification and detection was performed using a LightCycler DNA Master SYBR Green I kit (Roche Applied Science, Mannheim, Germany) in a LightCycler Detection System (Roche Applied Science). The PCR reaction was performed as follows: one cycle of 95°C for 10 minutes and 40 cycles of 95°C for 15 seconds, 60°C for 20 seconds and 72°C for 15 seconds. The primer sequences were as follows: β-actin, 5′-TCCTGTGGCATCCACGAAACT-3′ (forward) and 5′-GAA GCATTTGCGGTGGACGAT-3′ (reverse); NCL, 5′-AACCTCTCCTACAGTG CAACA-3′ (forward) and 5′-CTGGCTTCTGGCATTAGGTG-3′ (reverse).

### Resected HCC specimens

A total of 147 HCC paraffin specimens were collected by surgical resection at Department of Pathology at Kaohsiung Chang Gung Memorial Hospital from January 1987 to December 1998. The closing date of follow-up was 31 December 2006. All the HCC patients were diagnosed with resectable tumour(s) after liver biochemical test, and complete imaging studies including sonography, computed tomography, and/or angiography. The durations of follow-up were estimated in months. The survival of patients who died owing to HCC-unrelated factors during the long periods of follow-up was estimated till the event and treated as censored data. Hepatitis markers, serum alpha-fetoprotein (αFP) levels, and other clinical parameters of HCC patients were also recognized. All the HCC specimens consisted of both the tumour and adjacent non-tumour parts. Tumour sizes were recorded as the largest diameter in the specimen. The background of the non-tumour part was characterized as cirrhotic or non-cirrhotic. The differentiated states of HCC were divided into three groups as well (grade I carcinoma of Edmondson-Steiner classification), moderate (grade II carcinoma of Edmondson-Steiner classification), and poor (grades III and IV carcinoma of Edmondson-Steiner classification). The pathologic stages of HCC were classified according to the staging system by the International Union against Cancer with a minor modification: stage I, encapsulated, without evidence of liver or vascular invasion; stage II, unencapsulated or capsulated and with liver invasion, but without vascular invasion; stage III, invasion of small vessels in the tumour capsule or focal invasion of portal vein branches close to the tumour; and stage IV, invasion of portal vein in the distal liver (1 cm away from the tumour capsule), branches of major portal vein, common bile duct, or perforation into visceral peritoneum. The experimental protocols were approved by the institutional review committee.

### Immunohistochemical analysis

Immunohistochemical analysis of NCL expression in HCC samples was performed using primary antibodies against NCL (1:500 dilution; Sigma-Aldrich) and detected by colorimetric reaction using a HRP-linked polymer detection system (Biogenex; San Ramon, CA) with counterstaining by Gill's hematoxylin. The nuclear and cytoplasmic NCL staining in HCC and non-neoplastic counterpart were graded, respectively. The slides were scanned with Pannoramic Scan (3D HISTECH, Budapest, Hungary) for grading of NCL nuclear staining. Briefly, three representative areas in either HCC or non-neoplastic tissue were analyzed with ImmunoScreener and NuclearQuant (3D HISTECH) and graded as none or weak, intermediate and strong staining after software analysis. Finally, HCC tissues with weak or intermediate NCL staining were combined as low group (*n* = 107) and compared with strong NCL staining (labeled as high; *n* = 40) for subsequent correlation between the NCL LI and clinicopathological parameters of HCC, as well as Cox hazard regression model.

### NCL RNA interference

NCL gene silencing was achieved though transfection with NCL siRNA using the siRNA transfection reagent according to the manufacturer's instructions (Santa Cruz and Life Technologies). The sequences of the pool in NCL siRNA#1 were as the following: sense 1: CUACGGCUUUCAAUCUCUUtt and antisense 1: AAGAGAUU GAAAGCCGUAGtt; sense 2: UGUUGUGGAUGUCAGAAUUtt and antisense 2: AAUUCUGACAUCCACAACAtt; sense 3: CCUGUGGUCUCCUUGGAAAtt and antisense 3: UUUCCAAGGAGACCACAGGtt, and sense 4: UGAUAGAGCUAA CCCUUAUtt and antisense 4: AUAAGGGUUAGCUCUAUCAtt (Santa Cruz). The sequences of NCL siRNA#2 were as the following: sense, 5′-GCGGAGAUCAGAUUAGUCAtt-3′ and antisense, 5′-UGACUAAUCUG AUCUCCGCag-3′ (Life Technologies, Austin, TX). For each transfection, 0.5 μg of NCL siRNA or scramble siRNA with 4 μL of siRNA transfection reagent was added to siRNA transfection media. The siRNA transfection media was overlaid onto the cells for 6 hours. The media was then aspirated and the cells were maintained in DMEM supplemented with FCS for the further study. After transfection for 48 hours, hepatoma cells were subjected to various functional assays in the absence or presence of HDGF (10 ng/ml) for indicated time interval.

### Cell proliferation assay

The cells were incubated in DMEM supplemented with HDGF (10 ng/mL) or the indicated reagents. After 20 hours incubation, the media was added 3-[4,5-dimethylthiazol-2-yl]-2,5-diphenyl-tetrazolium bromide (MTT) solution for additional 4 hours. Subsequently, cells were lysised with isopropanol supplemented with 50 mM hydrochloride, and the absorbance at 570 nm was measured.

### Cell invasion assay

The cell invasion was measured using the trans-well assay in a Boyden chamber (48-well plate) using a polycarbonate filter (8 μm pore size; Nucleopore, Costar, Cambridge, MA). The polycarbonate filter was coated with Matrigel (2 mg/mL; BD Biosciences) at 4°C for 1 hour and placed on the lower compartment of a 48-well plate with DMEM containing 10% FCS as a chemoattractant. The cells (1 × 10^5^ cells/mL) supplemented in DMEM containing HDGF (10 ng/mL) or the indicated reagents were seeded in the wells (50 μL/well) of the upper compartment. After 24-hours of incubation, the invading cells on the lower surface of the polycarbonate filter were stained with 0.5% crystal violet and observed by microscopy. The number of invading cells was counted from at least four high-power fields (200 × magnification) per assay, and the data are presented as the averages of triplicate experiments.

### Colony formation assay

Flat colony formation assay was performed as previously described [5]. For NCL overexpression or gene silencing, the cells were harvested by trypsinization at 24 hours after transfection with NCL-GFP or NCL siRNA, and then seeded in DMEM supplemented with 0.5% FCS. The colony number was counted after fixing with paraformaldehyde and staining with crystal violet in 10% buffer formalin.

### Statistical analysis

Data were expressed as the mean ± standard deviations (SD) of at least three independent experiments. Comparisons between groups of independent samples were assessed by the student's *t*-test, one-way ANOVA, Mann-Whitney *U* test or the Kruskal-Wallis test. The associations between categorical variables were assessed using the Chi-square test or Fisher's Exact test. Survival rates were calculated by the Kaplan-Meier methods and the difference in survival was compared with the log-rank test. The influence of various clinicopathological features on overall survival was assessed by the Cox proportional hazard model. A *P*-value < 0.05 was considered statistically significant.

## SUPPLEMENTARY FIGURES AND TABLES



## Abbreviations

HCC: hepatocellular carcinoma
HDGF: hepatoma-derived growth factor
NCL: nucleolin
PI3K: phosphatidylinositol 3-kinase
αFP: alpha-fetoprotein
siRNA: small interfering RNA
LC-MS/MS: liquid chromatography tandem mass spectrometry
co-IP: co-immunoprecipitation
qRT-PCR: quantitative reverse transcription-polymerase chain reaction
