# Supplementary material for: Hepatoma-derived growth factor/nucleolin axis as a novel oncogenic pathway in liver carcinogenesis
Source: Oncotarget. 2015 Apr 17;6(18):16253–70. doi: 10.18632/oncotarget.3608 (PMC4599268; doi:10.18632/oncotarget.3608)
Supplement: Supplementary file 1 [file oncotarget-06-16253-s001.pdf]

SUPPLEMENTARY FIGURES AND TABLES

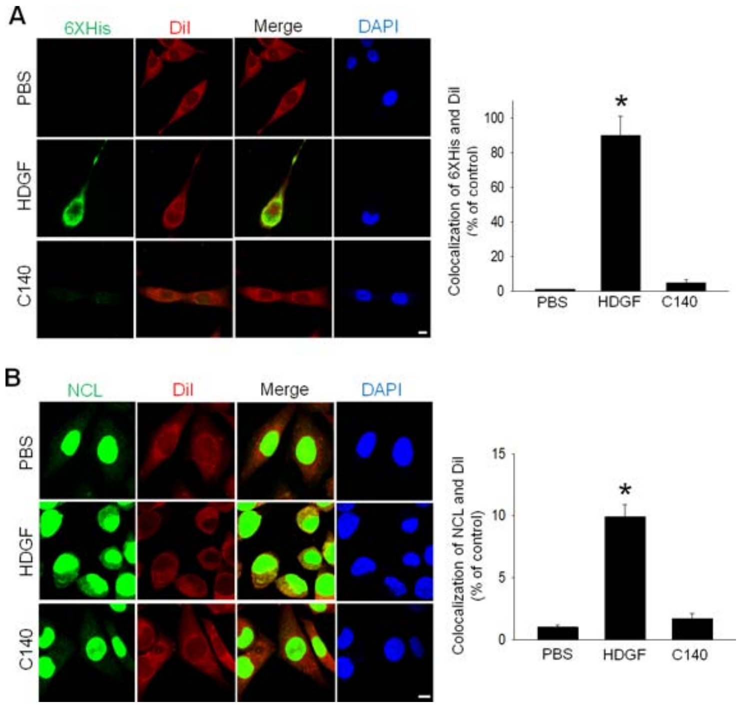

Supplementary Figure S1: Effect of exogenous HDGF on NCL distribution in SK-Hep1 cells. A. After treatment with exogenous HDGF and C140 (10 ng/mL) for 4 hours, cells were permeabilized by Triton X-100 and stained with anti-6xHis (green) and DiI dye (red). Bar, 20 µm. The quantification of colocalization of 6xHis staining and DiI staining at the cytoplasmic membrane of SK-Hep-1 cells. **B.** After treatment with exogenous HDGF and C140 (10 ng/mL) for 4 hours, cells were permeabilized by Triton X-100 and stained with anti-NCL (green) and DiI dye (red). Bar, 20 µm. The quantification of colocalization of NCL staining and DiI staining at the cytoplasmic membrane of SK-Hep-1 cells. Data were normalized to the control group and presented as the mean ± SD as percentages of the control. *$P < 0.05$ versus control.

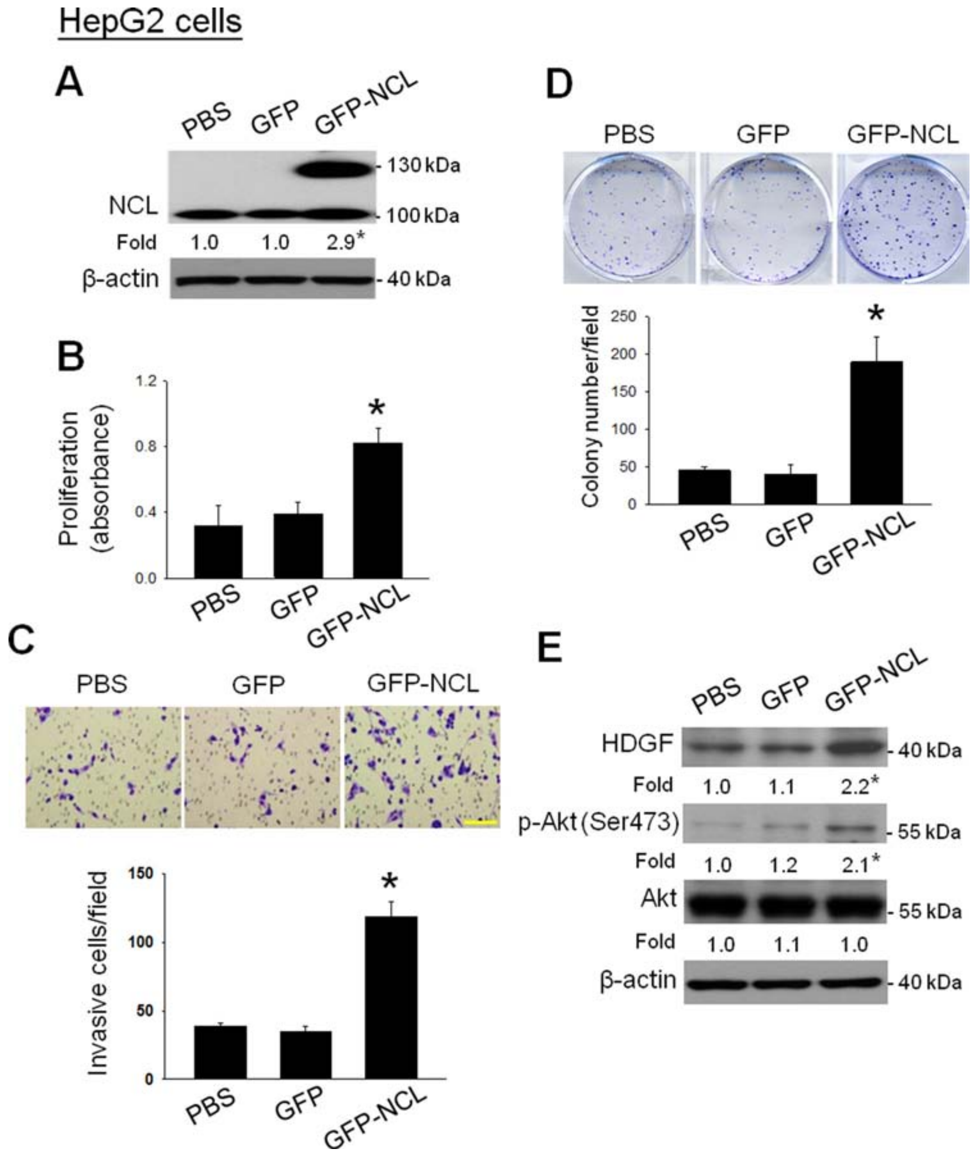

Supplementary Figure S2: Influence of NCL overexpression on the oncogenic behaviours and HDGF/Akt signaling of hepatoma HepG2 cells. After transfection with pEGFP-C1 vector encoding NCL fused with green fluorescent protein (NCL-GFP) for 48 hours, cells were harvested for subsequent analysis. **A.** Effect of NCL-GFP transfection on NCL expression in HepG2 cells. The protein level of NCL was assessed by immunoblot assay. **B.** Effect of NCL-GFP transfection on proliferation of HepG2 cells. Cell proliferation was assessed by MTT assay. **C.** Effect of NCL-GFP transfection on invasion of HepG2 cells. The invasion capability was assessed by Boyden chamber assay. Bar, 250 µm. **D.** Effect of NCL-GFP transfection on anchorage-independent growth of HepG2 cells. The colony-forming capability was determined by crystal violet and counted. **E.** Effect of NCL-GFP transfection on HDGF expression and Akt activation in HepG2 cells. The protein levels of HDGF and p-Akt were assessed by immunoblot assay. The data are presented as the mean ± SD as percentages of the control. *$P < 0.05$ versus control.

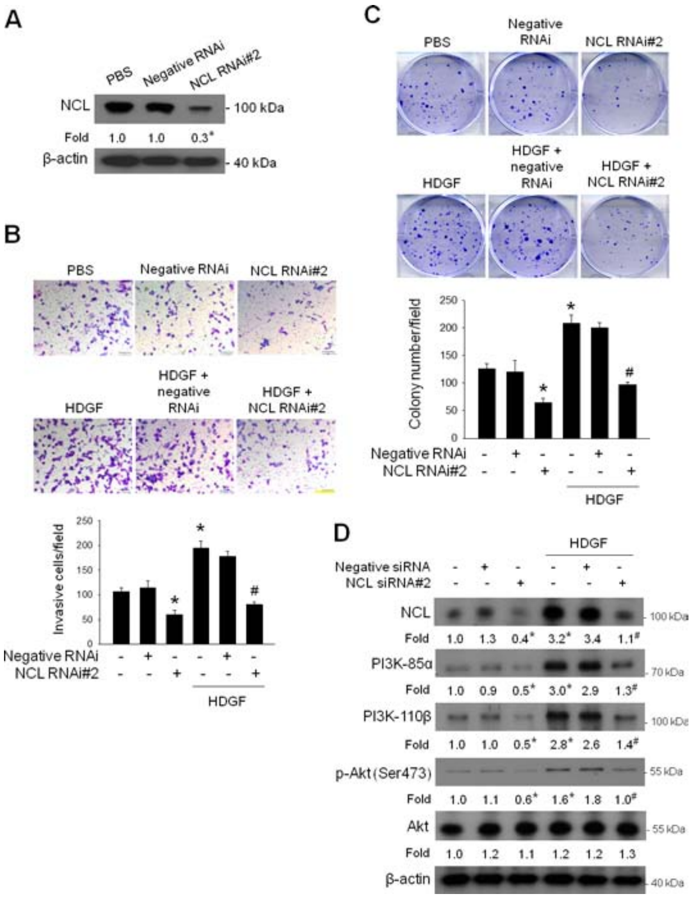

Supplementary Figure S3: Effect of NCL knockdown on the basal and HDGF-stimulated oncogenic behaviours and PI3K/Akt signaling of hepatoma SK-Hep-1 cells. After treatment with HDGF (10 ng/mL) in the absence or presence of NCL siRNA#2 (0.5 μg) for the indicated times, cells were harvested for the subsequent analysis. **A.** Effect of NCL siRNA#2 on NCL protein level in SK-Hep-1 cells. After transfection for 72 hours, NCL expression was detected by immunoblot analysis using an anti-NCL antibody. **B.** Effect of NCL siRNA#2 on invasiveness of SK-Hep-1 cells. After treatment with HDGF for 24 hours, the invasion capability was assessed by Boyden chamber assay. Bar, 250 μm. **C.** Effect of NCL siRNA#2 on anchorage-independent growth of SK-Hep-1 cells. After treatment with HDGF for 10 days, the colony-forming capability was detected by crystal violet solution and counted. **D.** Effect of NCL siRNA#2 on PI3K/Akt signaling of SK-Hep-1 cells. After treatment with HDGF for 24 hours, the protein levels of NCL, PI3K-85α, PI3K-110β, p-Akt and Akt were assessed by immunoblot assay. The data are presented as the mean ± SD as percentages of the control. $*P < 0.05$ versus control. $^{\#}P < 0.05$ versus 10 ng/mL HDGF.

Supplementary Table S1. MS/MS Analysis of NCL Protein

Accession number[1)]	Protein name	Protein molecular weight /pI	Score[2)]	Number of unique peptides matched to protein sequence	% Sequence coverage[3)]	Subcellular location[4)]
gi|189306	Nucleolin	76.355 / 4.59	948	21	32%	Nucleolus, Nucleus, Cytoplasm, and Plasma membrane

[1)]NCBI accession number of the identified protein.
[2)]Mascot score of the identified protein.
[3)]% Sequence coverage of matched peptides in the identified protein.
[4)]Subcellular locations were referred to the Human Protein Reference Database (http://www.hprd.org/).

Supplementary Table S2. The Matched Peptides within Human NCL Sequence

NCL residues	Sequence	Charge state	Predicted mass	Observed mass
296 – 318	K.QKVEGTEPTTAFNLFVGNLNFNK.S	3	2550.2701	2551.2595
298 – 318	K.VEGTEPTTAFNLFVGNLNFNK.S	2	2310.4382	2311.1485
325 – 333	K.TGISDVFAK.N	2	936.3952	936.4917
334 – 342	K.NDLAVVDVR.I	2	1001.4028	1000.5189
348 – 362	R.KFGYVDFESAEDLEK.A	3	1775.6515	1775.8254
349 – 362	K.FGYVDFESAEDLEK.A	2	1647.7096	1647.7304
363 – 370	K.ALELTGLK.V	2	843.4298	843.5065
371 – 382	K.VFGNEIKLEKPK.G	3	1400.6533	1400.8027
404 – 420	K.VTQDELKEVFEDAAEIR.L	3	1990.837	1990.9847
411 – 420	K.EVFEDAAEIR.L	2	1177.42	1177.5615
430 – 444	K.GIAYIEFKTEADAEK.T	2	1683.8298	1683.8355
438 – 449	K.TEADAEKTFEEK.Q	2	1396.558	1396.6358
458 – 467	R.SISLYYTGEK.G	2	1159.4522	1159.5761
487 – 508	K.TLVLSNLSYSATEETLQEVFEK.A	3	2500.2481	2500.2584
524 – 537	K.GYAFIEFASFEDAK.E	2	1593.7112	1593.7351
524 – 545	K.GYAFIEFASFEDAKEALNSCNK.R	3	2510.1499	2511.1264
555 – 561	R.LELQGPR.G	2	811.4018	811.4552
578 – 589	K.GLSEDTTEETLK.E	2	1321.5288	1321.6249
578 – 597	K.GLSEDTTEETLKESFDGSVR.A	2	2198.6086	2199.0179
611 – 624	K.GFGFVDFNSEEDAK.E	2	1560.624	1560.6733
625 – 645	K.EAMEDGEIDGNKVTLDWAKPK.G	3	2345.086	2345.1209
